# Social and health-related predictors of family function in older spousal caregivers: a cross-sectional study

**DOI:** 10.1590/1980-57642020dn14-040007

**Published:** 2020-12

**Authors:** Sofia Cristina Iost Pavarini, Allan Gustavo Bregola, Bruna Moretti Luchesi, Déborah Oliveira, Fabiana de Souza Orlandi, Fernanda Gomez de Moura, Helena Rita Oliveira Silva, Nathalia Alves de Oliveira, Marisa Silvana Zazzetta, Ariene Angelini dos Santos-Orlandi, Ana Carolina Ottaviani

**Affiliations:** 1 Department of Gerontology, Universidade Federal de São Carlos – São Carlos, SP, Brazil.; 2 Graduate Program in Nursing, Universidade Federal de São Carlos – São Carlos, SP, Brazil.; 3 School of Health Sciences, University of East Anglia – Norwich, United Kingdom.; 4 Graduate Program in Nursing, Universidade Federal de Mato Grosso do Sul – Três Lagoas, MS, Brazil.; 5 School of Medicine, Department of Psychiatry, Universidade Federal de São Paulo – São Paulo, SP, Brazil.; 6 Universidade do Vale do Paraíba, Graduate Program in Gerontology – São José dos Campos, SP, Brazil.; 7 Private Practice – São Joaquim da Barra, SP, Brazil.; 8 Department of Nursing, Universidade Federal de São Calos – São Carlos, SP, Brazil.

**Keywords:** caregivers, aged, family relations, cognitive dysfunction, depression, emotional stress, cuidadores, idosos, relações familiares, disfunção cognitiva, depressão, estresse emocional

## Abstract

Given the benefits of adequate family function for the health and well-being of older adults, it is important to understand what factors predict adequate family function in older people who care for their spouses.

**Objective:**

Analyse predictors of family function in older spousal caregivers.

**Methods:**

A cross-sectional study design was used to investigate a non-probabilistic sample of 298 older spousal caregivers. Home-based face-to-face interviews were used to evaluate sociodemographic variables and care context, family function (Family APGAR), cognitive function, perceived stress, and depressive symptoms. Data were analysed using multiple logistic regression with stepwise forward method for variable section.

**Results:**

Older caregivers having some degree of cognitive impairment (OR=-0.160, 95%CI 0.444–0.579), depressive symptoms (OR=-0.848, 95%CI 0.726–0.992) or high levels of stress (OR=-0.955, 95%CI 0.914-0.999) had overall lower levels of family function. Having more children was linked to approximately 1.3 times higher family function (95%CI 1.080–1.057).

**Conclusion:**

Stress, depression, cognitive decline, and number of children are predictors of family function and should be considered in social and health care strategies within the family caregiving context.

## RESUMO.

Dados os benefícios da função familiar adequada para a saúde e o bem-estar de pessoas idosas, é importante compreender quais fatores predizem o funcionamento familiar adequado em pessoas idosas que cuidam de seus cônjuges.

**Objetivo:**

Analisar preditores de funcionamento familiar em idosos cuidadores de cônjuges idosos.

**Métodos:**

Um estudo transversal foi usado para investigar uma amostra não probabilística de 298 cuidadores cônjuges idosos. Foram realizadas entrevistas domiciliares para avaliar variáveis sociodemográficas e situação de cuidado, funcionamento familiar (APGAR familiar), função cognitiva, percepção de estresse e sintomas depressivos. Os dados coletados foram analisados por meio de regressão logística múltipla com método *stepwise forward* para seleção das variáveis.

**Resultados:**

Idosos cuidadores com algum grau de comprometimento cognitivo (OR=-0,160, IC95% 0,444–0,579), sintomas depressivos (OR=-0,848, IC95% 0,726–0,992) ou altos níveis de estresse (OR=-0,955, IC95% 0,914-0,999) tiveram menores níveis de funcionamento familiar. Ter mais filhos esteve relacionado a maiores níveis de funcionamento familiar em aproximadamente 1,3 vez (IC95% 1,080–1,057).

**Conclusão:**

A presença de estresse, depressão, declínio cognitivo e número de filhos são preditores do funcionamento familiar e devem ser considerados como parte de estratégias sociais e de saúde no contexto de cuidado familiar.

## INTRODUCTION

The increase in life expectancy of the population combined with the high prevalence of chronic diseases is related to an increase in functional capacity limitations in the older adults.^1^ These limitations require long-term care, which mostly come from the family and informal context.^1^ ^,^ ^2^ Current family arrangements offer intergenerational support and provide the opportunity for older couples to live together longer.^3^ In such cases, it is common for older individuals to become responsible for the care provision of their dependent older spouses, especially older women.

Family functionality is described as the dynamics of family relationships that are formed within families throughout their history,^4^ with adaptation, companionship, affection and ability to resolve the family with its members.^5^ Family function can potentially affect the social, emotional, and physical health of individuals.^6^ It reflects the family's ability to meet the essential life goals of its members and the way the family members interact with each other within the family unit.^7^ A family with adequate levels of family function is more likely to solve critical situations with emotional stability. Conflicts are often solved in a more balanced way, without overloading any family member, and individuals are able to adapt harmoniously in response to changes in life and stressful events.^8^

High demands for informal care, as well as changes in day-to-day routines and social roles, may lead to high levels of perceived burden and stress in family caregivers,^9^ which are associated with lower levels of perceived and received support,^10^ as well as poor family function.^11^ When the family struggles to adapt to the challenges arising from ageing and the presence of chronic diseases, family relationships may be affected negatively, impacting the physical, emotional, and psychological well-being of older adults.^12^ ^,^ ^13^

Considering a qualitative approach, studies indicate that when the impairment of functional capacity affects the elderly, the entire family system is also affected, regardless of social class^14^ ^,^ ^15^. Regarding the caregiver, the family support provided to the elderly can occur in an imposed way, without emotional/social support and information about the care to be provided. The lack of specific knowledge and preparation of families for the care causes negative changes in social support and family relationships^15^.

Previous meta-analyses have shown that lower levels of social support are associated with higher levels of perceived burden on family caregiver and with poorer cognitive performance in older adults,^16^. Older people may receive support from different sources, but family members are the major source^17^ Sustaining good quality family function helps maintain the health and well-being of those older people who provide care for dependent family members.^2^ Whilst being female and experiencing high family demands can cause high levels of strain, having fewer family demands, a stronger bond with family members, and having better education as well has been shown to be associated with better family function.^18^

A previous study with 2,052 Brazilian older adults demonstrated that poor cognitive function and higher dependence, as well as not having children, were predictors of low family function. However, living with someone else, as opposed to alone, was found to be an important predictor of adequate family function. The authors indicated that in old age, the lack of autonomy, dependence, dementia, and missing social support, affect the quality of life of the elderly. It is believed that the presence of family members increases the safety of the elderly, since they can assist in daily activities and also contribute to social development.^19^

The literature has shown a rapid increase in the number of older people involved with family caregiving worldwide, and it has been argued that more needs to be done to support these people.^20^ ^,^ ^21^ Previous meta-analysis has pointed out that spousal caregivers are more likely to be older, providing more hours of care a day and for many years, and are living in poorer physical health conditions when compared to younger, children, or in-law caregivers.^22^

Given the benefits of adequate family function for the health and well-being of older adults, it is important to understand whether sociodemographic aspects and the context of care, cognitive function, depressive symptoms and perceived stress predict adequate family function in older people who are caring for their spouse. These variables have not been evaluated in previous studies in this population. The current study aimed to identify some of the predictors of family function in older individuals providing care for their spouses. We anticipate that this will help researchers and clinicians plan and deliver effective family interventions aimed at better supporting these individuals in Brazil.

## METHODS

This study used a cross-sectional exploratory design with a convenience sample. Participants were living in the city of São Carlos-SP, Brazil, and met the following inclusion criteria: 1) be aged ≥60; 2) be registered at one of the primary health care services of Sao Carlos, SP; 3) be the primary caregiver for a spouse aged ≥60 and dependent on care for at least one basic activity of daily living (BADL) or at least one instrumental activity of daily living (IADL) as assessed by the Katz Index^23^ and the Lawton and Brody Scale;^24^ and 4) be willing to provide informed consent. The exclusion criteria were: 1) both older spouses in the family were dependent for BADL and IADL; 2) older adults showed severe listening or visual impairments that compromised their ability to respond to the questionnaires; and 3) candidates had sufficient communication difficulties to prevent their understanding of the questions.

Participants were recruited via patient registries within each of the primary health care services in Sao Carlos, SP. A total of 594 families were initially identified, 26 were excluded because of death of one of the older partners, 28 because of change of address, and 69 for not being found at their address after three contact attempts. A total of 471 families were visited and 84 refused to take part in the study. The older couples had their functional capacity evaluated (BADL and IADL) and 36 were excluded for being both dependent on care. Of the 351 remaining potential participants, 53 were non-spousal caregivers and were excluded, totalling the final sample of 298 older spousal caregivers. Trained researchers collected informed consent forms and interviewed the participants at their home after a first contact. Each interview was carried out once and lasted approximately one hour and thirty minutes.

All ethical procedures for research with people were respected, following the 466/2012 National Brazilian Resolution, regimented by the National Health Council. The project was authorised by the city health council and was approved by the Research Ethics Committee at the Universidade Federal de São Carlos (416.467/2013). Data were collected after the participant read, understood, and provided informed consent. The researchers ensured that the individuals who were interested in taking part in the study met the inclusion criteria and were cognitively capable of providing consent.

### Measurement outcomes

- Sociodemographic and care information: these data were collected using a questionnaire created by the research team and contained the following variables: gender (female/male), age (years), schooling (years), family income (in relation to national minimum wage), number of children (continuous), number of years of caregiving (years), amount of time caregiving per day (hours), material/financial and compassionate/emotional support received to care (yes/no), level of dependence of the person cared for (BADL — Katz Index^23^ and IADL — Lawton and Brody Scale).^24^
- Family function (Family APGAR):^2^ This is a commonly used tool to measure family function through the evaluation of individuals’ satisfaction in relation to five areas: adaptation, partnership, growth, affection and resolution. It helps identify whether family function is adequate (13+ points), moderately dysfunctional (9–12 points) or highly dysfunctional (1–8 points).^25^ ^,^ ^26^ A previous integrative review investigating the use of Family APGAR scores to evaluate family relationships in older adults showed that this instrument is easy to use and interpret, allowing the detection of family dysfunction in older adults and their caregivers.^27^
- Cognitive function (Addenbrooke's Cognitive Examination Revised — ACE-R):^28^ ^,^ ^29^ this is a brief battery assessing five domains: orientation/attention, memory, verbal fluency, language and visual-spatial skills. Total scores range from zero to 100 points, with the higher the scores, the better the cognitive performance, where the threshold for each domain is defined as follows: <17 points for orientation/attention, <15 points for memory, <8 points for verbal fluency, <22 points for language and <13 visual-spatial skills. For the purpose of data analysis, participants had their total scores divided into two groups, one below and the other above the median (median=64). The ACE-R were translated into Brazilian Portuguese and had good reliability and validity for the cut-off proposals.^30^
- Depressive symptoms (Geriatric Depression Scale — GDS-15):^31^ ^,^ ^32^ the total scale scores vary from zero to 15, 0–5 means absence of depressive symptoms, 6–10 means mild depressive symptoms, and 11–15 means severe depressive symptoms. The GDS-15 has been translated for use in Brazil and represents the classic assessment to evaluate mood in older people.
- Perceived stress (Perceived Stress Scale):^33^ ^,^ ^34^ this scale is composed of 14 items. The total scores result from the sum of all the responses and range from zero to 56, and the higher the total score, the higher the perceived stress levels. PSS has been translated to Brazilian Portuguese and reports good internal consistency in its application in older people for the full version of the assessment (α=0.82).^34^

### Data analysis

Data analysis was carried out in *Statistical Package for the Social Sciences* (SPSS®), version 22. Basic descriptive statistics were calculated for all the studied variables (frequency, means, medians and standard deviations). Multiple logistic regression using a stepwise forward method for variable selection was used to identify the predictors of family dysfunction.^35^ The dependent variable was family function based on the Family APGAR scores (1=poor family function or 2=adequate family function). The continuous independent variables were the following: number of children (continuous), perceived stress levels (total scores) and depressive symptoms (total scores). The caregivers’ cognitive status was considered to be a dichotomous variable divided into two categories: 1=with cognitive impairment, 2=without cognitive impairment, following the values above or below the median threshold. A threshold level of p<0.20 was used for the variable section for the univariate analyses. To have a good performance of regression model, a criteria decision to variable selection was adopted with a threshold level of p<0.20 in univariate analyses. For the multivariate analysis, a threshold of p<0.05 was used.

## RESULTS

The 298 older spousal caregivers taking part in the study were mostly females (78.2%), with a mean age of 69.9 (±6.9) years old and most between 60 and 69 years old (54.4%). The mean schooling degree was of 3.5 (±3.2) years and 63.1% had at least incomplete primary school. The mean number of children was 4.3 (±2.7) and 52.7% had 4 children or more. The mean family income was of R$2,266.29 (approximately $677.89 USA dollars), with 55.0% reporting more than 3 minimum salary units (approximately $650.51 USA dollars). With regard to the care provided, 43.3% reported being caregivers for more than five years, and the majority (62.4%) provided up to five hours of care a day for their spouses. About 85% reported that they did not receive any material or financial help, and almost 54% indicated that they did not receive any compassionate/emotional support (Table 1).

**Table 1:** Sociodemographic and care information (n=298), São Carlos, SP, Brazil, 2014.

		n	%
Gender			
	Female	233	78.2
	Male	65	21.8
Age (years)			
	60–69	162	54.4
	70–79	100	33.5
	≥80	36	12.1
Schooling (years)			
	None	58	19.4
	1–4	188	63.1
	5–8	28	9.4
	≥9	24	8.1
Family income (in Brazilian minimum salary units)*			
	Up to 1 minimum salary	35	11.8
	2–3	99	33.2
	>3	164	55.0
Number of children			
	0–3	139	46.6
	≥4	157	52.7
	Information not provided	2	0.7
Years of caregiving			
	<1	57	19.1
	1–5	100	33.6
	>5	129	43.3
	Information not provided	12	4.0
Hours per day caregiving			
	≤5	186	62.4
	6–9	37	12.4
	≥10	66	22.1
	Information not provided	9	3.1
Material/financial help received			
	No	254	85.3
	Yes	43	14.4
	Information not provided	1	0.3
Compassionate/emotional support			
	No	160	53.7
	Yes	136	45.6
	Information not provided	2	0.7

* Brazilian minimum salary (1 minimum salary unit=R$ 724,00 or U$216.92 in the first semester of 2014).

With regard to family function, 85.6% (n=255) reported adequate family function, 7.4% (n=22) moderate family function and 7.0% (n=21) poor family function. The mean scores for the cognition measurement tool (ACE-R) was 62.9 (±18.4) and the mean scores for each domain were as follows: attention/orientation=13.5 (±2.9), memory=14.6 (±6.2), verbal fluency=5.9 (±2.9), language=18.3 (±5.6), and visual-spatial skills=10.3 (±3.6). Approximately 50% all spousal caregivers scored lower than the expected overall median for the ACE-R. The majority of the sample (77.8%, n=228) did not have depressive symptoms and 19.1% (n=56) had mild depressive symptoms, while 3.1% (n=9) had severe symptoms. With regard to perceived stress levels, the mean was of 18.52 (±10.7) points (moderate to low).

Table 2 shows the results from the multiple logistic regression analysis. The results demonstrated that family function decreased approximately 0.95 for every point increase in the perceived stress scale. Similarly, having some degree of cognitive impairment (OR=0.160, 95%CI 0.444–0.579) or presence of depressive symptoms (OR=0.848, 95%CI 0.726–0.992) also decreased family function to some degree. Having more children was shown to increase family function by approximately 1.3 times (95%CI 1.080–1.057).

**Table 2:** Multiple logistic regression investigating the predictors of family dysfunction in older spousal caregivers (n=298), São Carlos, SP, Brazil, 2014.

	OR	p-value	95%CI
Perceived stress	-0.955	0.045	0.914–0.999
Cognitive impairment	-0.160	0.005	0.444–0.579
Depressive symptoms	-0.848	0.039	0.726–0.992
Number of children (continuous)	1.276	0.004	1.080–1.057

OR: *Odds Ratio*; 95%CI: 95% confidence interval.

## DISCUSSION

This study investigated the role of cognitive, emotional and sociodemographic variables as possible predictors of family function in caregivers of older spouses. The results showed that high levels of stress and depressive symptoms and presence of cognitive impairment predicted low levels of family function. Having a higher number of children predicted increased levels of family function.

The use of a cross-sectional design may not allow for drawing conclusions about whether such variables may predict family function over time. In addition, the use of a non-probabilistic sample may not allow for the generalization of the findings to the wider population of spousal caregivers. However, our findings can potentially help improve an understanding of the importance of developing and establishing health and social care policies for older caregivers aimed at improving family function and informal care management. It also contributes to the international literature around family caregiving in later life, which is currently lacking.

In the present study, a large proportion of our sample were women who reported adequate family function. Approximately 50% scored lower than the expected overall median for the ACE-R, did not have depressive symptoms and perceived moderate to low stress levels. Previous studies report that caregivers, especially those of an individual with dementia or who experience a decline in cognitive performance or increase burden and care-related stress.^36^ ^–^ ^38^ For family functionality, the values found in the present study with the Family Apgar instrument were in line with what was found with elderly people in the interior of São Paulo^39^ and Chile.^40^

This is important because women also often receive low levels of support, have more physical impairment, have lower income, and experience higher levels of stress and burden.^41^ Previous studies attempting to explain the relationships between the variables investigated in this study and family function in family caregivers are somewhat limited, but there is evidence suggesting a potential bidirectional relationship between family function and psychological well-being in older adults. For example, a study carried out with 304 North Korean adults with high risk for depression found that family function and resilience were predictors for the development of depressive symptoms;^42^ however, no study that tried to explain this relationship with caregivers was found.

There is relative consensus in the literature that informal/family support network is very important for the mental health and cognition of older adults.^16^ ^,^ ^43^ ^,^ ^44^ A systematic review of 39 studies suggests a relationship between social activity and global cognition, overall executive functioning, working memory, visuospatial abilities and processing speed. In addition, benefits older adults’ cognitive functioning and changes in the characteristics of social relationships could be a consequence of cognitive decline as opposed to a cause however.^16^ Previous studies have suggested that low frequency of interactions, small social network size, or negative experiences of social support predict more rapid cognitive decline.^45^ ^,^ ^46^

Previous Brazilian research conducted with older adults found that those experiencing family dysfunction were more likely to report the presence of depressive symptoms compared to those without such dysfunction.^47^ The current study demonstrated that such associations can be bidirectional, with better cognitive health and less depressive symptoms predicting better family function in older people who are caregivers. The interaction between family relationships and health is bidirectional, since the worsening of health status leads to a restriction of the social network, while a decrease in social networks, in a repeated and prospective way, predicts serious morbidities and mortality.^48^ This is important as it has been pointed out in previous studies that older people who are caregivers are more likely to report the presence of depressive symptoms and more cognitive impairment than older people who are not caregivers.^38^

During adult life, one of the partners may take more responsibility in the family day-to-day activities. In later life, this person may be in need of care and this dynamic may need to change with the other person taking over the family responsibilities. Such changes in the family dynamics often generate insecurity, anger, resentfulness, and guilt, which can lead to or increase family conflicts.^49^ ^–^ ^51^ When the number of children is higher and the existing social support network around the older caregiver is strong, the likelihood of such feelings arising is reduced, which in turn helps protect the family function and the well-being of the older adults involved in the caregiving dyad.^52^

The current study did not find significant associations between family function and other individual and care-related variables that have been considered relevant to the population of older caregivers in the literature. For example, previous studies have shown that high levels of perceived burden, stress and depression are prevalent in older caregivers^53^ and are often associated with poor education and longer hours providing care.^49^ These, consequently, have been associated with lower family function.^51^ This study presented clear associations of factors with dysfunctional family, which could mean that these findings were linked to the specific sample study and could not be assumed for a general population. This study can support the development of protocols in health units and home care programs to track family functionality and intrinsic and extrinsic care resources, training of professionals working with the elderly segment and the insertion of professionals trained in gerontology in health teams, the design of monitoring programs for caregivers as psychoeducational groups, gerontological services (day centres, caregiver community centres, expansion of home care programs in Brazil), and strengthening of social and family support networks, in addition to educational work on ageing and the realities of care in communities, as indicated in the Global Plan for Attention to Dementia established by the World Health Organization., with goals between 2017 and 2025, with the creation of dementia-friendly communities.^54^

Having fewer children and also high stress levels, depressive symptoms and cognitive impairment were found to be predictors of family dysfunction in a Brazilian sample of older spousal caregivers. Research and support services for family caregivers are limited in Brazil and are mostly focused on reducing burden and stress only. The current study results demonstrated the importance of maintaining the cognitive health and psychological well-being of older caregivers, as well as considering the family arrangements for support to promote better family function for these individuals.

Future research should focus on studying a larger prospective cohort of older caregivers to understand the long-term impact of such factors on the older caregivers’ physical and mental health.
